# Intermediate uveitis: pattern of etiology, complications, treatment and outcome in a tertiary academic center

**DOI:** 10.1186/s13023-017-0638-9

**Published:** 2017-04-27

**Authors:** Thomas Ness, Daniel Boehringer, Sonja Heinzelmann

**Affiliations:** Eye Center, Medical Center, University of Freiburg, Faculty of Medicine, Killianstr. 5, 79106 Freiburg, Germany

**Keywords:** Intermediate uveitis, Etiology, Systemic associations, Complications, Treatment

## Abstract

**Background:**

Patients with intermediate uveitis (IU) represent a heterogenous group characterized by a wide spectrum of etiologies and regional differences. Aim of the study was to analyze the characteristics of patients with IU examined in an academic center in Germany.

**Methods:**

We conducted a retrospective analysis of the clinical records of all patients with intermediate uveitis referred to the Eye Center, University of Freiburg from 2007 to 2014. Diagnosis followed the Standardization in Uveitis Nomenclature (SUN) criteria. Data analysis included: etiology of IU, demographics, complications, treatment and visual acuity.

**Results:**

We identified 159 patients with intermediate uveitis during that period. Mean age at diagnosis was 35 years. Most are female (64%), and the mean duration of IU was 6.1 years (range 1 month – 35 years). Etiology of IU was idiopathic in 59%. Multiple sclerosis (MS) (20%) and sarcoidosis (10%) were frequent systemic causes of IU. Other etiologies including infectious diseases (tuberculosis, borreliosis) or immune-mediated conditions (eg, after vaccination) were present in 11%. The pattern of complications included macular edema (CME) (36%), cataract (24%), secondary glaucoma (7%), and epiretinal membrane formation (19%). Periphlebitis and optic neuritis were more frequent in conjunction with MS. Treatment comprised local and systemic steroids, immunosuppressive agents, biologics, and surgery. Best corrected visual acuity was better than 20/25 in 60% of the eyes after more than 10 years of follow-up.

**Conclusions:**

In our German academic center, most IU cases were idiopathic or associated with MS or sarcoidosis. In contrast to other countries, infectious cases were rare. Patients’ overall visual prognosis is favorable even when the duration of IU has been long and and despite numerous complications.

## Background

Uveitis in general and especially intermediate uveitis (IU) fulfills the criteria as a rare disease, and the National Institutes of Health (NIH) defines it as such [1, 2]. Uveitis experts standardized the nomenclature for uveitis (SUN) in 2005. According to these criteria, intermediate uveitis is defined as an intraocular inflammation mainly focused on the vitreous and peripheral retina [3, 4]. Intermediate uveitis accounts for 1.4 – 31% of all uveitis patients [1, 5–13]. The incidence of IU varies between 1.4 – 2/100.000 [9, 14, 15]. IU can be a sight-threatening disease and usually affects young adults [4, 9]. It is potentially associated with infectious and non-infectious diseases. Infectious diseases that may cause IU are tuberculosis, leprosy, Lyme’s disease, syphilis, toxocariasis, Whipple’s disease, and others. There is wide variation depending on specific geographic and cultural factors [4, 5, 12–14, 16]; eg, tuberculosis is more frequent in underdeveloped countries [17]. Associated systemic diseases are multiple sclerosis (MS), sarcoidosis and others [4, 9, 18]. Cases not of infectious origin or associated systemic disease are considered idiopathic. Clinical features are cellular inflammation of the vitreous, peripheral vascular sheathing, and the formation of snow balls or snow banks [4, 9]. The most common reason for loss of visual acuity is cystoid macular edema (CME). Other complications are cataract, epiretinal membranes, optic neuritis, and glaucoma [4, 9, 14, 16].

We conducted this study to analyze the demographic and clinical data of patients with IU in our academic center in Central Europe.

## Methods

This was a retrospective study including all patients with intermediate uveitis examined at the Eye Center, University of Freiburg between 2007 and 2014. Intermediate uveitis was classified according to recommendations by the SUN working group [3].

Our study received institutional review board approval (EK Freiburg 19/15). Patient consent was not required as this was a retrospective, pseudoanonymous chart review. Patients diagnosed with any disorder other than intermediate uveitis were excluded. All patients were examined in a specialized uveitis center and treated in a multidisciplinary setting. If necessary, the appropriate specialists were consulted to determine any suspected underlying systemic or infectious disease.

In the case of sarcoidosis we collected chest radiographs, computer tomographies, bronchoalveolar lavage results, biopsies and laboratory data, if available. Diagnosis of infectious IU was based on serological testing and systemic manifestations, if applicable.

Data analysis included: etiology of IU, demographics, complications, treatment modalities, visual acuity and final outcome. Continous factors are presented as mean, standard deviation, standard error of the mean and confidence interval. Categorial data are presented as percentages. We used chi-sqare statistics for hypothesis testing. Change in visual acuity is presented as Box- and Whisker Plot.

All calculations were performed with the R-platform using only core functionality [19].

## Results

During the study period we identified 159 patients suffering from IU. Their mean age varied from 5 to 80 years (mean 35.2 years; standard deviation (SD) 17.1; standard error of the mean (SEM) 1.35; 95% confidence intervall (CI) ± 2.66). Mean follow-up was 6.1 years (SD 6.9; SEM 0.55; CI ±1.08) (Table 1).

**Table 1:** ᅟ

Demographic data	
Gender Female (n (%))	102 (64%)
Age at diagnosis (years) (mean/SD/SEM/CI)	mean 35.3 SD 17.1 SEM 1.35 CI ± 2.66
Follow up (years) (mean/SD/SEM/CI)	mean 6.1 SD 6.9 SEM 0.55 CI ± 1.08
Etiology (n = patients (%))	
Idiopathic	93 (58.3%)
MS	31 (19.5%)
Sarcoidosis	16 (10%)
Infectious	6 (4%)
Miscellaneous	13 (8%)
Initial visual acuity (logMar) (mean/SD/SEM/CI) eyes	mean 0.2 SD 0.43 SEM 0.02 CI ± 0.05
Final visual acuity (logMar) (mean/SD/SEM/CI) eyes	mean 0.14 SD 0.33 SEM 0.02 CI ± 0.04
Initial visual acuity >20/25 (n (%))	211 (66%)
Final visual acuity >20/25 (n (%))	241 (75%)

**Fig. 1:**
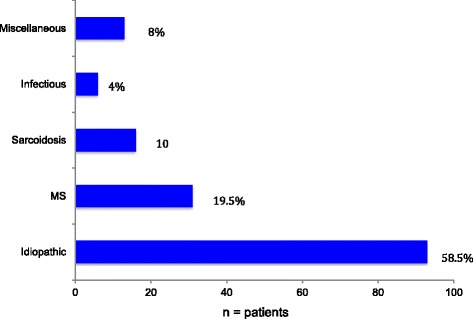
Etiology of IU (*n* = number of patients)

Nearly two-thirds of these patients were female (64%). The duration of IU at the date of inclusion in the study varied from 1 to 420 months (mean 73 months; SD 83; SEM 6.6; CI ±12.9). Regarding the etiology, 58.5% of the IU cases were idiopathic. Multiple sclerosis accounts for 19.5% and sarcoidosis for 10% of the patients (*n* = 16) (definite ocular sarcoidosis *n* = 2, presumed ocular sarcoidosis *n* = 5 and probable ocular sarcoidosis *n* = 9 according to IWOS (International Workshop of ocular sarcoidosis) criteria [20]). Various infectious diseases like Lyme’s disease (*n* = 5) or tuberculosis (*n* = 1) were detected in 4%. None of these patients was immunocompromised. Other underlying diagnosis summarized under the term miscellaneous were made in 8% of the IU patients (Fig. 1). In detail, the miscellaneous group comprised cases with post immunization (FSME) (*n* = 1), juvenile idiopathic arthritis (JIA) (*n* = 3), psoriasis (*n* = 4), fibromyalgia (*n* = 1), Behcet’s disease (*n* = 2); Crohn’s disease (*n* = 1), and vemurafenib therapy (*n* = 1).

**Fig. 2:**
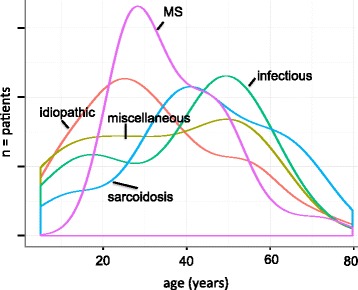
Age distribution of the different etiologies of IU (*n* = number of patients)

The age at diagnosis varied with the underlying origin of IU. Patients with idiopathic IU were the youngest (mean 32.9 years (SD 17.7; SEM 1.8; CI ± 3.6), followed by the miscellaneous group (mean 34.7 years; SD 18.9; SEM 5.2; CI ± 10.2). Patients with sarcoidosis (mean 44.1 years; SD 17.6; SEM 4.4; CI ± 8.6), MS (mean 36.9 years; SD 12.6; SEM 2.3; CI ± 4.4) and infectious diseases (mean 39.0 years; SD 3.3; SEM 1.3; CI ± 2.6) were older at the time of diagnosis. The distribution of age at the time of diagnosis is shown in Fig. 2. In patients with an infectious origin, there is a peak in patients under 20 years of age, and another in those about 50 years of age.

**Fig. 3:**
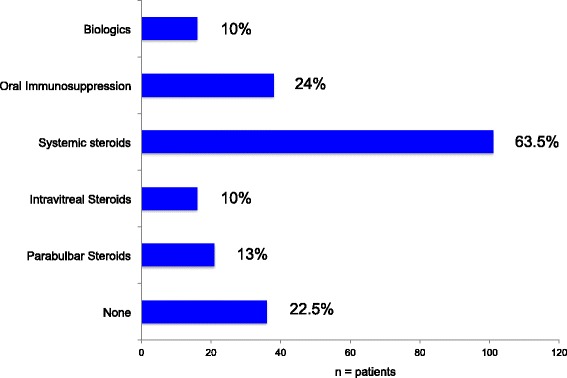
Therapy of IU (oral immunosuppression: AZA, MTX, MMF, CsA) (*n* = number of patients)

Only 22.5% of the IU patients required no systemic or parabulbar treatment. Most received systemic steroids (63.5%), intravitreal steroids (10%), or parabulbar steroids (13%). Systemic immunosuppression (azathioprine, methotrexate, mycophenolate mofetil or cyclosporine A) was necessary in 24%. Biologics were used in 10% (mainly interferon alpha) (Fig. 3). The main indications for initiating therapy are summarised in Table 2. Some patients got more than one therapy. Usually we started treatment with oral, parabulbar or intravitreal steroids. If there was no stable remission with less than 7.5 mg prednisolon equivalent, an immunosuppressive or biologic agent was added.

**Table 2:** Indication for therapy (*n* = patients)

	Steroids			Oral immunosuppression	Biologic
	parabulbar	intravitreal	systemic		
CME	12	11	50	19	16
Optic neuritis	0	0	7	0	1
Vitreous inflammation	12	2	59	24	4
Underlying disease	0	0	9	6	5

**Fig. 4:**
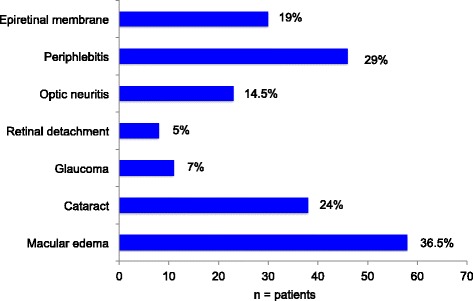
Complications of IU (*n* = number of patients)

A total of 62% of the IU patients developed at least one complication. Cystoid macular edema was the most frequent complication (36.5%). Nearly a quarter suffered from cataract (23.9%), 19% from epiretinal membrane, 5% from retinal detachment, and 7% from glaucoma (Fig. 4). Periphlebitis and optic neuritis were significantly related to MS-associated IU (*p* < 0.001 Chi Square Test).

**Fig. 5:**
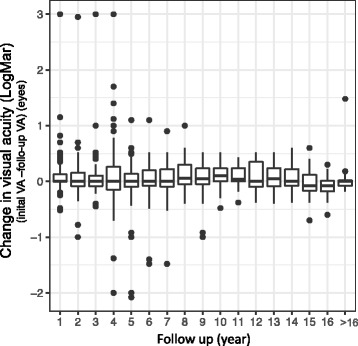
Difference in visual acuity at different time points after diagnosis (initial visual acuity (LogMar) – visual acuity at follow-up (years) (LogMar) (*n* = eyes) (Box- and Whisker Plot (median; 1^St^ and 3^rd Quartile^; range))

**Fig. 6:**
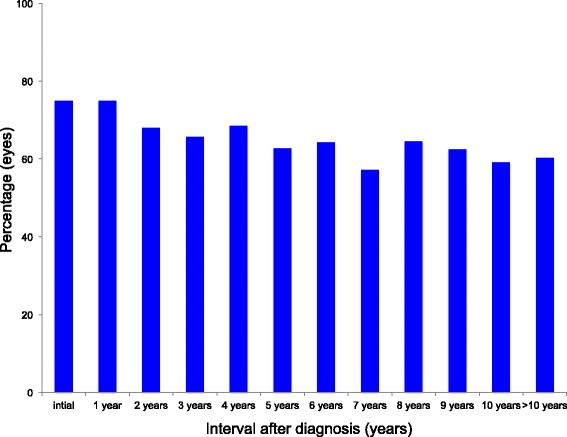
Percentage of eyes with a visual acuity better than 20/25 at different time points after diagnosis (*n* = eyes)

The overall prognosis was favorable. As Fig. 5 illustrates, visual acuity was stable over time in most patients. At the end of follow-up, 75% of the eyes had a best corrected visual acuity better than 20/25 (Table 1). As shown in Fig. 6, the percentage of eyes with visual acuity of 20/25 or better was slightly decreasing with follow-up. After a follow up of at least 10 years more than 60% fulfilled this criterium.

## Discussion

Our study demonstrates that IU in Central European patients is mostly non-infectious and idiopathic, requiring therapy in 80% of cases, and that it has an overall favorable prognosis. However, many patients experience at least one of many complications (eg. cataract, glaucoma, CME, epiretinal membrane). Many of these patients fulfilled the criteria for the older term pars planitis, which is restricted by SUN for “that subset of intermediate uveitis associated with snowbank or snowball formation in the absence of an associated infection or systemic disease” [3].

Like in our cohort, most other researchers have noted that IU usually affects young adults. The mean age at diagnosis varies between 22.6 and 33 years of age [14, 16, 21–23]. In contrast to other studies, we differentiated age by etiology. We observed a marked difference in age at diagnosis depending on the underlying disease. The youngest patients suffered from idiopathic IU, the oldest from infectious IU. In addition, we detected in conjunction with infectious IU a biphasic age distribution, with one peak in children and a second one in the fifth decade.

In Europe, the US and China, IU is usually idiopathic [1, 9, 11, 13, 14, 16]. In contrast, in other parts of the world such as India, there is a marked proportion of infectious IU rising to 58% [24]. In these countries, tuberculosis is a very common comorbidity; as the cause of IU in Europe and US it is rare [17]. On the other hand, MS is a frequent underlying disease in IU in our patients and in the US [14]. The proportion of MS in IU patients varies from 7 to 30.4% [1, 21, 25–31]. In our cohort, MS was very significantly associated with periphlebitis, a particular indication of IU. Others have observed the same [21, 32]. Since IU might be the first manifestation of MS and early treatment seems to improve the overall prognosis, it is important to screen all IU patients for MS [33–35].

About 10% of our patients have sarcoidosis, a diagnosis that is also frequent elsewhere in the world [1, 9, 11, 16, 31, 36]. We have found that soluble interleukin 2 receptor is a useful screening parameter [37].

Immunization as a cause of IU is currently under discussion. As described by several groups, FSME immunization may trigger IU in some patients [38–42]. JIA is associated with anterior uveitis. Our three cases suffered from IU and the pediatricians found no other underlying disease than JIA.

Many IU patients suffer from complications. The development of cataract, glaucoma, CME, epiretinal membrane formation, retinal detachment, periphlebitis or optic neuritis is similar worldwide [4, 9, 10, 14, 30, 43]. Cataract and glaucoma might be caused by IU itself or by treatment of IU, especially with corticosteroids. There is ample evidence that CME and epiretinal membrane formation correlate with poor visual prognosis [4, 9, 16].

As in the smaller study by Donaldson et al, nearly 2/3 of our patients required therapy [14]. Main treatment indications in our series were CME or severe vitreous inflammation. Systemic, intraocular and parabulbar corticosteroids are the predominant therapeutic options. Only a quarter of our patients received immunosuppressive agents – more frequently than in China and the US [8, 14, 16].

Regarding our cohort’s MS patients: about 10% received biologics, mainly interferon.

Despite the many complications, IU’s overall prognosis is encouraging. Most patients have retained best corrected visual acuity of 20/40 or better [4, 14, 16, 43]. The decrease of visual acuity during follow-up in our study might be biased by the fact, that patients with no complications and good visual acuity were lost for follow up. In our specialized center, those with complications were followed for a longer time.

Our study is limited by its retrospective character. Nevertheless, we report on a large number of patients and have delivered useful data for daily clinical practice.

## Conclusions

In our German academic center, most IU cases were idiopathic or associated with MS or sarcoidosis. In contrast to other countries, infectious cases were rare. Patients’ overall visual prognosis is favorable even when the duration of IU has been long and and despite numerous complications.

## Abbreviations

CME: Cystoid macular edema
EK: Ethikkommission
FSME: Frühsommermeningoenzephalitis
IU: Intermediate uveitis
JIA: Juvenile idiopathic arthritis
MS: Multiple sclerosis
NIH: National Institutes of Health
SUN: Standardization of uveitis nomenclature
US: United States
